# Supplementary material for: Complexome profiling of the Chlamydomonas psb28 mutant reveals TEF5 as an early PSII assembly factor
Source: Plant Cell. 2025 Mar 17;37(6):koaf055. doi: 10.1093/plcell/koaf055 (PMC12164590; doi:10.1093/plcell/koaf055)
Supplement: koaf055_Supplementary_Data [file koaf055_supplementary_data.zip › psb28_interactive.zip!/plots/intensity/qualityPlots/peptides/EIF4A_Cre06.g298650.t1.2.html]

### Cre06.g298650.t1.2

Trivial name: EIF4A  
SeqCoverage [median]: 10.30  
Mass: 52.86 kDa  
  
 

| Sequence | MissedCleavage | Mass [Da] | wtIntensity | muIntensity | Score |
| --- | --- | --- | --- | --- | --- |
| ELAQQIEK | 0 | 957.51 | 0.00 | 462.60 | 87.18 |
| GIYAYGFEKPSAIQSK | 1 | 1757.90 | 0.00 | 1507.83 | 39.01 |
| GLDVIQQAQSGTGK | 0 | 1400.73 | 0.00 | 483.66 | 119.39 |
| VLITTDLLAR | 0 | 1113.68 | 8400.17 | 0.00 | 130.31 |
|  |  |  | 8400.2 | 2454.1 |  |
